# The cGAS-STING signaling pathway in the regulation of pulmonary infections: a systematic review

**DOI:** 10.3389/fcimb.2025.1628481

**Published:** 2025-07-08

**Authors:** Qinchao Xu, Jiayu Zhu

**Affiliations:** Department of Pediatrics, Shaoxing Central Hospital, The Central Affiliated Hospital of Shaoxing University, Shaoxing, Zhejiang, China

**Keywords:** cGAS-SING, innate immune, immune escape, lung infection, type I interferons

## Abstract

The lungs are constantly exposed to airborne pathogens and depend on robust innate immune surveillance for protection. The cyclic GMP–AMP synthase (cGAS)–stimulator of interferon genes (STING) signaling pathway, a core component of the innate immune system, plays a pivotal role in defending against respiratory infections caused by viruses, bacteria, and mycobacteria, including *Mycobacterium tuberculosis*. Dysregulation of this pathway has been linked to several chronic lung diseases, such as cystic fibrosis, chronic obstructive pulmonary disease (COPD), idiopathic pulmonary fibrosis, and asthma. Upon sensing cytoplasmic DNA, cGAS activates the STING pathway, producing type I interferons and pro-inflammatory cytokines that drive host immune response. However, many pathogens have developed strategies to evade detection or surpass cGAS-STING signaling. This systematic review highlights the molecular mechanisms governing cGAS-STING activation, its interaction with lung pathogens, and its potential as a therapeutic agent in respiratory diseases.

## 1 Overview

The innate immune system serves as the first line of defense against invading pathogens. Host cells detect pathogen-associated molecular patterns (PAMPs) through pattern recognition receptors (PRRs), initiating signaling cascades that induce the production of type I interferons (IFN-I) and pro-inflammatory cytokines, which promote pathogen clearance (Akira et al., 2006; Hu and Shu, 2018). Recent studies have demonstrated that the cyclic GMP–AMP synthase (cGAS)–stimulator of interferon genes (STING) signaling pathway plays a crucial role in immune responses and various other cellular processes, including autophagy, translation regulation, metabolic homeostasis, cell aggregation, DNA damage repair, cellular senescence, and programmed cell death (Chen and Xu, 2023).

**Figure 1:**
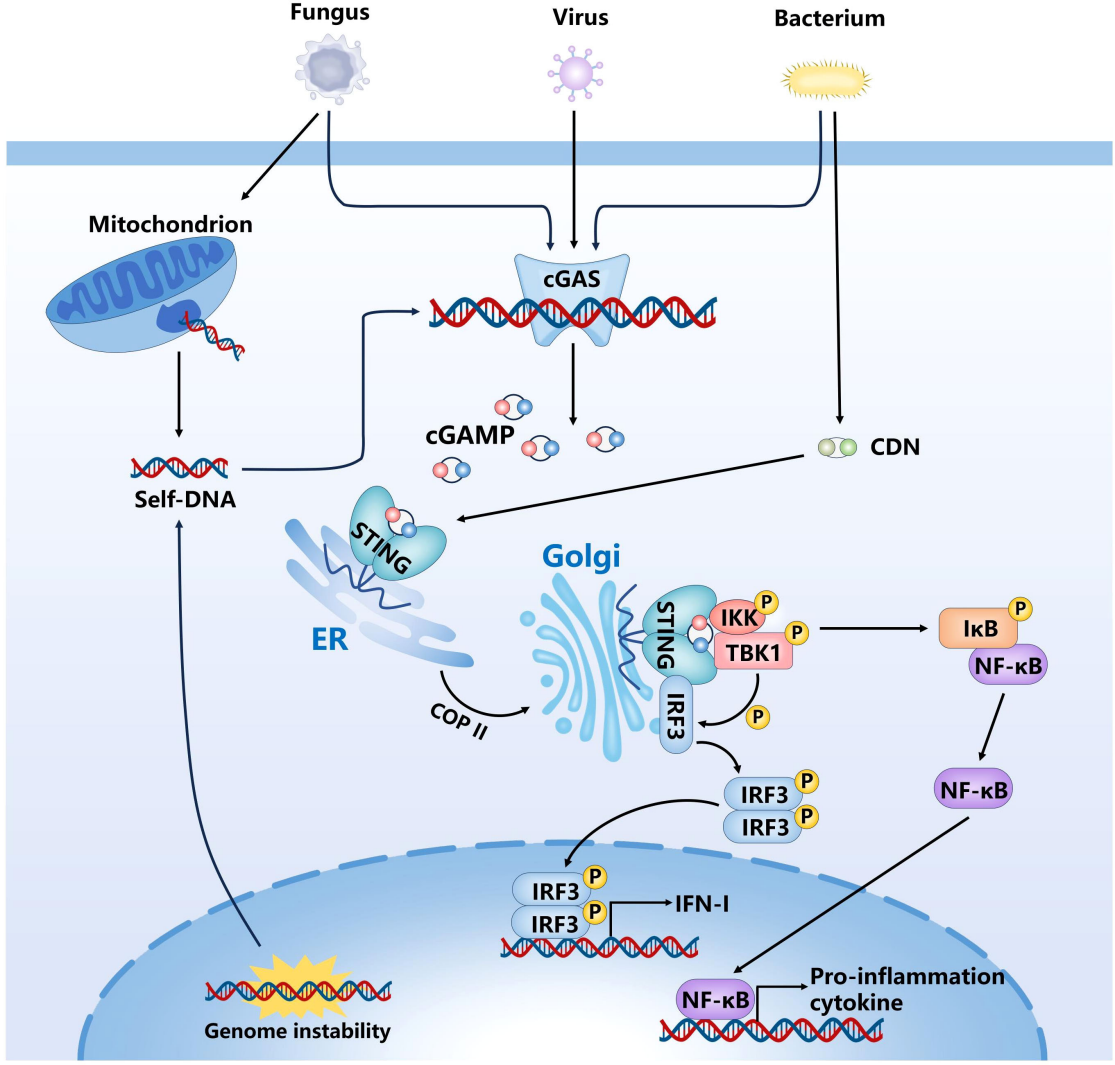
Activation of the cGAS-STING signaling pathway in response to cytosolic DNA. cGAS recognizes dsDNA in the cytoplasm and catalyze cGAMP. Subsequently, cGAMP binds to and activates STING, and promotes its transport from ER to Golgi apparatus, STING initiates downstream signaling cascades by activating TBK1-IRF3 and NF-κB pathway.

The cGAS-STING signaling axis constitutes a pivotal innate immune surveillance system, enabling host cells to recognize aberrant cytoplasmic DNA and mount an effective antiviral/antimicrobial defense response (**Figure 1**). Upon detecting double-stranded DNA (dsDNA) in the cytoplasm, cGAS— a key cytosolic DNA sensor—recognizes DNA derived from pathogens, such as DNA viruses, retroviruses, bacteria, and parasites, as well as from host sources like nuclear and mitochondrial DNA (mtDNA) (Ablasser and Chen, 2019; Hopfner and Hornung, 2020). It binds dsDNA via its positively charged surface, forming droplet-like condensates and catalyzing the synthesis of the second messenger 2’3’-cGAMP from GTP and ATP (Ablasser et al., 2013; Wu et al., 2013). The cGAMP ligand generated in the process subsequently interacts with STING, an adaptor protein resident in the endoplasmic reticulum (ER). The binding of cGAMP to STING induces conformational changes that facilitate its oligomerization and translocation from the ER to the Golgi apparatus via the Coat Protein Complex II (COPII)-dependent vesicular trafficking pathway (Dobbs et al., 2015; Gui et al., 2019). Once in the Golgi lumen, activated STING functions as a scaffold for the recruitment of downstream signaling molecules, such as TANK-binding kinase 1 (TBK1) and interferon regulatory factor 3 (IRF3) (Liu et al., 2015; Zhang et al., 2019). Phosphorylation of IRF3 by TBK1 promotes its nuclear translocation, where it serves as a transcriptional activator for IFN-I genes (Li et al., 2013). Upon secretion, IFN binds to IFN receptor 1 (IFNAR1) on the cell surface via autocrine or paracrine mechanisms, activating the JAK-STAT pathway. This activation promotes the formation of the IFN-Stimulated Gene Factor 3(ISGF3)complex, consisting of STAT1, STAT2, and IRF9, which subsequently translocate into the nucleus and binds to the interferon-stimulated response element (ISRE), thereby initiating the transcription of interferon-stimulated genes (ISGs) (Stark and Darnell, 2012). ISGs maintain an antiviral state through a variety of mechanisms, including inhibition of viral entry and membrane fusion, degradation of viral nucleic acids, blockade of viral replication and transcription, regulation of cell death, and modulation of immune responses.

The cGAS–STING axis can also facilitate the activation of both the canonical and noncanonical nuclear factor-kappa B (NF-κB) signaling pathways, thereby further enhancing the expression of pro-inflammatory cytokines and chemokines. The canonical NF-κB pathway is typically activated in response to various pro-inflammatory stimuli, such as tumor necrosis factor-alpha (TNF-α), interleukin-1β (IL-1β), or PAMPs, and involves the activation of the IκB kinase (IKK) complex (Karin and Ben-Neriah, 2000). This complex comprises three subunits: IKKα, IKKβ, and the regulatory subunit NEMO (IKKγ) (Zandi et al., 1997; Yamaoka et al., 1998). STING has been shown to interact with tumor necrosis factor receptor-associated factor 6 (TRAF6), which leads to the recruitment and activation of both TBK1 and the IKK complex (Abe and Barber, 2014). Activation of IKK results in phosphorylation and subsequent degradation of IκBα, thereby releasing NF-κB dimers—primarily the p65/p50 heterodimer—from inhibition and allowing their translocation into the nucleus to initiate transcription of inflammation-related genes (Traenckner et al., 1995). The noncanonical NF-κB pathway is mainly activated by a subset of specific receptors, including CD40, B-cell activating factor receptor (BAFF-R), and lymphotoxin β receptor (LTβR) (Futterer et al., 1998; Claudio et al., 2002), and relies predominantly on the accumulation of NF-κB-inducing kinase (NIK). Stabilized NIK activates IKKα homodimers, which phosphorylate the p100 precursor, leading to its partial degradation into p52 and the formation of p52/RelB heterodimers that translocate into the nucleus to regulate transcription of specific target genes (Solan et al., 2002). Although the cGAS–STING pathway primarily activates the canonical NF-κB signaling cascade, it may, under certain conditions, indirectly influence the noncanonical NF-κB pathway by modulating NIK stability, altering the function of TRAF proteins, or shaping a chronic inflammatory microenvironment. Notably, in contrast to the canonical pathway, the noncanonical NF-κB pathway is independent of IKKβ and NEMO, exhibits a slower activation kinetics, and is primarily involved in lymphoid organogenesis, B cell maturation, and long-term immune regulation. In addition, STING can enhance NOD-like receptor protein 3 (NLRP3) expression through the NF-κB signaling pathway and directly facilitate the assembly of the NLRP3 inflammasome. Upon activation, the NLRP3 inflammasome cleaves caspase-1, thereby initiating Gasdermin D (GSDMD)-mediated pyroptosis and promoting the maturation and secretion of IL-1β and IL-18, ultimately amplifying the inflammatory response (Gaidt et al., 2017; Luo et al., 2024). Taken together, the dual signaling outputs highlight the pivotal role of the cGAS-STING pathway in coordinating both antiviral and inflammatory defense mechanisms, ensuring cellular homeostasis against diverse microbial threats.

In addition to the canonical signaling pathways, the cGAS-STING pathway regulates various cellular processes through non-canonical mechanisms. One such pathway is the recently identified STING-PERK-eIF2α axis (Zhang et al., 2022), in which STING interacts with protein kinase R (PKR)-like ER kinase (PERK) at the ER to activate eIF2α, thereby regulating mRNA translation and plays a critical role in cellular senescence and fibrotic diseases. Furthermore, STING activation can induce autophagy via a TBK1-independent mechanism, contributing to antiviral defense by degrading damaged cellular components and invading pathogens (Gui et al., 2019). Autophagy is a highly conserved intracellular degradation process essential for immune defense, including pathogen clearance, regulation of inflammation, antigen presentation, and lymphocyte homeostasis (Deretic et al., 2013). The cGAS-STING pathway facilitates the efficient clearance of cytoplasmic DNA and viruses through autophagy induction and interferon-signaling.

The activity of the cGAS-STING pathway is regulated through multiple mechanisms. Tripartite motif-containing 29 (TRIM29), an E3 ubiquitin ligase, is expressed in various tissues and has been shown to play a pivotal role in modulating the cGAS-STING pathway, IFN production, and innate immune responses associated with viral infections. Mechanistically, TRIM29 attenuates cGAS-STING signaling by mediating K48-linked ubiquitination and degradation of STING, thereby suppressing the expression of IFN-I and pro-inflammatory cytokines. This ultimately promotes persistent infection by DNA viruses such as Epstein-Barr virus (EBV) and facilitates tumor immune evasion (Xing et al., 2017). Conversely, TRIM29 deficiency significantly enhances host resistance to Herpes simplex virus type 1 (HSV-1) and adenovirus infections, indicating that TRIM29 functions as a viral-exploited negative regulator of the cGAS-STING-TBK1-IRF3 axis, diminishing its antiviral capacity. In a viral myocarditis model (Wang et al., 2024), TRIM29 promotes PERK SUMOylation to stabilize PERK protein, leading to sustained ER stress, apoptosis, and reactive oxygen species (ROS) production. Excessive ROS can oxidize thiol residues in TBK1, inhibiting its dimerization and activation, which subsequently blocks downstream IFN-I induction via the STING pathway. In lung studies, TRIM29 is highly expressed in alveolar macrophages (AMs) and acts as a critical negative regulator by targeting NEMO for ubiquitination and degradation, thereby suppressing NF-κB and IRF3 pathway activation and reducing the expression of inflammatory mediators such as IFN-β and IL-6 (Xing et al., 2016). As a host factor exploited by viruses, TRIM29 plays a vital role in tissue-specific immune regulation. Targeting TRIM29 may represent a promising strategy for modulating innate immune responses and treating viral infections and their associated complications.

Other than cGAS, several other intracellular DNA sensors also contribute to the recognition aberrant DNA and activation of the innate immune responses. These include DNA-dependent activator of IFN-regulatory factor (DAI), Interferon gamma-inducible protein 16 (IFI16), and DEAD-box helicase 41 (DDX41). All three promote IFN-I production through activation of the STING-TBK1-IRF3 signaling pathway (Takaoka et al., 2007; Stratmann et al., 2015; Hu et al., 2020). Meanwhile, Absent in Melanoma 2 (AIM2) triggers IL-1β expression by assembling inflammasomes (Burckstummer et al., 2009).

Recent studies have demonstrated that cGAS is not only present in the cytoplasm but also localizes to the nucleus, where it binds tightly to nucleosomes in an inactive state. This interaction is mediated by the histone H2A-H2B acidic patch, preventing self-DNA recognition and ensuring cellular homeostasis (Pathare et al., 2020; Dvorkin et al., 2024). Despite this regulation, cGAS can detect self-DNA derived from chromosomal instability, micronuclei formation, mtDNA release, and neutrophil extracellular traps (NETs) (Thompson et al., 2010; West and Shadel, 2017; Rongvaux, 2018; Apel et al., 2021; Krupina et al., 2021). Under physiological conditions, various nucleases—including DNase I, DNase II, and DNase III (TREX1)—degrade excess DNA to prevent cytoplasmic accumulation and aberrant cGAS activation (Yang et al., 2007; Stetson et al., 2008). However, when these nucleases are impaired or nuclear or mtDNA leak into the cytoplasm, cGAS—which lacks the ability to distinguish self from non-self DNA— can become activated, contributing to inflammatory and autoimmune diseases (Li et al., 2021). A notable example is the Aicardi-Goutières syndrome (AGS), a leukodystrophy caused by TREX1 mutations that result in chronic IFN-I production via sustained cGAS activation, leading to neuroinflammation and immune-mediated demyelination (Giroud et al., 1986; Crow and Manel, 2015). While cGAS-STING signaling is crucial for antiviral defense and immune regulation, its dysregulation is implicated in autoimmune diseases such as systemic lupus erythematosus (SLE) (Hagiwara et al., 2021). Abnormal apoptosis, necrosis, or NET formation (NETosis) is frequently observed in SLE patients (Darrah and Andrade, 2012). These processes result in the release of self-DNA into the cytoplasm, where it is recognized by cGAS. This recognition triggers STING-dependent IFN signaling, thereby initiating an inflammatory cascade. Research has demonstrated that the activation level of the cGAS-STING pathway in monocytes from SLE patients is significantly elevated and positively correlates with disease activity (Murayama et al., 2020). Furthermore, SLE patients often exhibit overexpression of IFN-I-related genes (Kato et al., 2018). The cGAS-STING pathway represents a critical mechanism for driving IFN-I synthesis. Persistent activation of this pathway induces dendritic cells (DCs) to secrete large amounts of IFN-α, which subsequently promotes B cell activation and autoantibody production, thus exacerbating the pathological progression of SLE (Gkirtzimanaki et al., 2018). Moreover, excessive activation of the cGAS-STING pathway can drive a cytokine storm, as observed in severe COVID-19. Tissue damage caused by severe acute respiratory syndrome coronavirus 2 (SARS-CoV-2) leads to the release of self-DNA, triggering cGAS-STING activation and overproduction of IFN-I and pro-inflammatory cytokines (Liu et al., 2022), which exacerbates lung tissue injury and multi-organ failure. Therefore, the cGAS-STING pathway serves a dual role in host defense and inflammatory disease pathogenesis.

## 2 The role of cGAS-STING pathway in modulating antiviral immune responses

The cGAS-STING pathway is a key mechanism through which host cells detect cytoplasmic DNA and initiate innate immune responses. It defends against viral infection by inducing IFN-I and activating autophagy-mediated antiviral mechanisms. However, viruses have developed various strategies to evade or suppress the antiviral effects of the cGAS-STING pathway (**Table 1**).

**Table 1:** The immune evasion mechanisms employed by different pathogens.

Types of pathogens	Pathogens	Immune escape mechanism	Reference
Virus	HSV-1	1. UL56 directly binds to cGAS, inhibiting its DNA sensing and enzymatic functions. 2. PRMT6 methylates STING and inhibits TBK1 and IRF3 phosphorylation.	(Zheng et al., 2022; Kong et al., 2024)
	SARS-CoV-2	1. ORF10 not only suppresses STING oligomerization but also disrupts the interaction between STING and TBK1. 2. Nucleocapsid protein suppresses the assembly of the cGAS-G3BP1 complex by competitively interacting with G3BP1. 3. ORF3a disrupts the interaction between STING and LC3, leading to the inhibition of autophagy.	(Han et al., 2022; Cai et al., 2023; Su et al., 2023)
	IAV	1. NS1 binds to mtDNA and inhibits cGAS-mediated immune recognition. 2. PB1-F2 promotes ROS production, thereby enhancing NLRP3 inflammasome activation and inducing cell death. 3. HA induces the ubiquitination and degradation of IFNAR1, thereby reducing the host’s sensitivity to IFN-I.	(Xia et al., 2015; Anastasina et al., 2016; Laghlali et al., 2020)
	Adenovirus	1. E1A inhibits the transcriptional activation of ISGs. 2. E1B suppresses the expression of IFN-induced transcripts. 3. VA RNA inhibits PKR-mediated translation suppression, consequently enhancing viral protein synthesis.	(Anderson and Fennie, 1987; Liao et al., 1998; Chahal et al., 2012)
	hMPV	IL-1β promotes viral replication during a window period prior to IFN-I production.	(Wu et al., 2024)
Bacterium	*S. aureus*	In skin infection models, the cGAS-STING antagonizes the TLR signaling pathway, suppressing the production of the key inflammatory cytokine IL-1β and neutrophil recruitment.	(Scumpia et al., 2017)
	*S. pneumoniae*	1. It employs Pde1/Pde2 to rapidly hydrolyze the c-di-AMP, preventing leakage of CDNs into the host cytoplasm. 2. EndA endonuclease degrades bacterial DNA and NETs.	(Zhu et al., 2013; Wooten et al., 2020)
	*L. pneumophila*	1. SdhA maintains the integrity of the LCV membrane and prevents the leakage of bacterial DNA. 2. SidE-mediated PR-ubiquitination interferes with the host’s intracellular transport system.	(Harding et al., 2013; Xie et al., 2023)
	NTHI	Phosphocholine modification of LOS alters surface structure, reduces immune recognition, and indirectly suppresses cGAS-STING activation.	(Clark et al., 2012)
	*Chlamydia*	*In vivo*, IFN-I may attenuate the host’s innate immune response by inducing macrophage apoptosis.	(Qiu et al., 2008)
	PA	ExoU induces cell death by compromising host cell membrane integrity, thereby indirectly attenuating the activation of the cGAS-STING pathway.	(Hardy et al., 2021)
	*M. tuberculosis*	1. MmsA promotes STING degradation via p62-mediated autophagy and suppresses IRF3 activation. 2. Mutations in specific genes frequently impact the functionality of the ESX-1 secretion system. 3. CdnP suppresses STING-dependent IFN-I responses by hydrolyzing bacterial c-di-AMP and host-derived 2’3’-cGAMP.	(Dey et al., 2017; Sousa et al., 2020; Sun et al., 2020)
Fungus	*Aspergillus fumigatus*	1. RodA conceals PAMPs within the cell wall to prevent their detection by the host’s PRRs. 2. GT induces apoptosis in both macrophages and neutrophils, and suppresses the activation of the NF-κB pathway.	(Carrion et al., 2013; Günther et al., 2024)

In DNA virus infections, viral genomic DNA acts as the primary PAMP, and cytoplasmic cGAS functions as a key sensor for recognizing dsDNA. HSV-1 stands as the pioneering DNA virus documented to trigger the activation of the cGAS-STING signaling cascade, both under *in vitro* experimental conditions and vivo biological systems (Wang et al., 2020). Upon recognizing HSV-1 dsDNA, cGAS catalyzes the synthesis of 2’3’-cGAMP, activating downstream STING signaling and ultimately inducing the expression of antiviral genes. To evade this immune response, HSV-1 employs multiple mechanisms that promote viral replication and pathogenicity. For instance, the HSV-1 envelope protein UL56 directly binds to cGAS, inhibiting its DNA sensing and enzymatic functions, thereby reducing cGAMP production and downstream antiviral signaling (Zheng et al., 2022). In addition, HSV-1 upregulates arginine methyltransferase 6 (Prmt6), which methylates STING and inhibits TBK1 and IRF3 phosphorylation, ultimately suppressing IFN-I production and promoting viral persistence (Kong et al., 2024).

As the causative agent of the COVID-19 pandemic, SARS-CoV-2 induces immune dysregulation and pathological inflammation in the host. Infection with SARS-CoV-2 leads to mitochondrial dysfunction and nuclear envelope rupture, releasing the nuclear and mtDNA into the cytoplasm, which activates the cGAS-STING pathway, promoting antiviral responses and inhibiting viral replication (Zhou et al., 2021; Liu et al., 2022). STING also exerts antiviral effects through autophagy-mediated clearance of damaged cellular components and pathogens (Gui et al., 2019). However, SARS-CoV-2 has evolved multiple mechanisms to suppress and undermine its activity. Studies have shown that SARS-CoV-2 open reading frame 10 (ORF10) anchors STING in the ER, preventing translocation to the Golgi apparatus and subsequent oligomerization, thereby blocking downstream signaling and reducing IFN-I production (Han et al., 2022). ORF10 also interferes with the STING–TBK1 interaction and impairs IRF3 phosphorylation and nuclear translocation, further suppressing antiviral gene expression (Han et al., 2022). In addition, SARS-CoV-2 nucleocapsid protein undergoes liquid-liquid phase separation in response to DNA stimulation, competitively binds to G3BP1 and disrupts the cGAS–G3BP1 complex, thereby inhibiting cGAS recognition of dsDNA and activation of IFN-I signaling (Cai et al., 2023). SARS-CoV-2 also targets STING-mediated autophagy: its open reading frame 3a (ORF3a) protein binds STING via a cysteine-rich domain, disrupting its interaction with LC3 and specifically inhibiting STING-induced autophagy without affecting IRF3-mediated IFN-I production (Su et al., 2023). Unlike proteins that interfere with autophagosome-lysosome fusion, ORF3 selectively blocks STING-triggered autophagy, facilitating viral replication. Notably, ORF3a has been shown to restore the replication of HSV-1 and EV-A71 viruses suppressed by the cGAS-STING (Su et al., 2023), highlighting its critical role in viral replication. By inhibiting cGAS-STING-mediated autophagy, SARS-CoV-2 enhances its replication and evades host innate immunity. The distinct immunomodulatory functions of ORF3a highlight its potential as a therapeutic target in COVID-19 treatment.

Influenza A virus (IAV), a global pandemic threat, is often associated with severe inflammation and tissue damage (Short et al., 2014). Although IAV is an RNA virus, it can indirectly activate the cGAS-STING pathway. The ion channel activity of the IAV M2 protein induces mitochondrial stress, causing mtDNA leakage into the cytoplasm via mitochondrial antiviral signaling protein. The released mtDNA activates cGAS and DDX41, promoting STING-dependent IFN signaling and enhancing antiviral defense. IAV also facilitates the intercellular transfer of cGAMP through connexin 43 gap junctions, amplifying STING-dependent responses between neighboring cells (Moriyama et al., 2019). However, IAV has evolved several strategies to evade immune recognition. Its non-structural protein 1 (NS1) binds mtDNA, inhibiting cGAS-mediated immune recognition and suppressing STING activation (Anastasina et al., 2016; Moriyama et al., 2019). PB1-F2, a viral protein, localizes to mitochondria and promotes reactive oxygen species (ROS) production, which activates NLRP3 inflammasomes and induces cell death—potentially affecting cGAS-STING signaling indirectly (Laghlali et al., 2020). In addition, hemagglutinin (HA) induces ubiquitination and degradation of IFNAR1, impairing IFN-I responsiveness by downregulating the JAK-STAT pathway and ISG expression (Xia et al., 2015). These mechanisms illustrate how IAV modulates innate immune response and highlight potential therapeutic strategies against influenza viruses.

Adenovirus is non-enveloped dsDNA virus commonly associated with respiratory diseases, conjunctivitis, and gastroenteritis. In children and immunocompromised individuals, these infections can induce severe, potentially life-threatening pulmonary diseases. Upon infection, adenoviral DNA is released into the cytoplasm, which is sensed by cGAS, activating the STING signaling pathway. Studies have demonstrated that cGAS and STING are critical in initiating anti-adenoviral responses, particularly in bone marrow-derived macrophages and dendritic cells (Anghelina et al., 2016). The adenoviral infection triggers an IRF3/IFN/ISGs antiviral response via the cGAS-STING pathway, and the intensity of the response varies depending on the adenoviral serotype and host cell type (Lam and Falck-Pedersen, 2014). Despite the cGAS-STING pathway activation, adenoviral replication remains largely unaffected (Lam and Falck-Pedersen, 2014), suggesting that adenoviruses employ immune evasion strategies to bypass antiviral response. Adenoviral gene products, including E1A, E1B, E3, E4, and VA RNA, have been implicated in dampening host immune responses (Mahr and Gooding, 1999; Burgert et al., 2002; Hendrickx et al., 2014). For example, E1A interferes with the transcriptional activation of ISGs (Anderson and Fennie, 1987; Reich et al., 1988), while E1B suppresses IFN-induced gene expression (Chahal et al., 2012). In addition, VA RNA inhibits PKR-mediated translation arrest, promoting viral protein synthesis (Liao et al., 1998). These evasion mechanisms predominantly target downstream immune signaling, allowing adenoviruses to circumvent the antiviral effects of cGAS-STING activation.

Human metapneumovirus (hMPV) is a critical pathogen associated with lower respiratory tract infections in children and immunocompromised individuals. In recent years, the role of inflammatory factors in hMPV infection has been extensively investigated, with particular focus on the IL-1β-mediated innate immune response. Notably, patients with severe hMPV infection exhibit significantly elevated levels of IL-1β, surpassing those observed in respiratory syncytial virus (RSV)-infected individuals (Malmo et al., 2016; Park et al., 2017). This enhanced expression demonstrates both time-dependent and dose-dependent characteristics and is positively correlated with hMPV replication (Wu et al., 2024). Accumulating evidence indicates that IL-1β not only contributes to inflammatory responses but also regulates viral replication via the cGAS-STING pathway, thereby influencing disease progression. Specifically, IL-1β induces the release of mitochondrial DNA (mtDNA), leading to increased cytoplasmic mtDNA enrichment and subsequent activation of the cGAS-STING signaling pathway (Aarreberg et al., 2019; Wu et al., 2023). While the cGAS-STING pathway is conventionally recognized for its role in promoting IFN-I-mediated antiviral defense, Wu et al. demonstrated that during the early stages of infection (within 72 hours), cGAS-STING activation paradoxically enhances hMPV replication (Wu et al., 2024). This finding was further supported by experiments using the cGAS inhibitor RU.521, which significantly restricted hMPV replication and abolished the enhancing effect of exogenous IL-1β on viral replication. These results suggest that IL-1β primarily promotes hMPV replication through the cGAS-STING pathway. The IL-1β-cGAS-STING axis may enhance viral replication during the pre-IFN-I production window, enabling evasion of host antiviral immune suppression.

## 3 The role of cGAS-STING pathway in mediating resistance to bacterial infections

Emerging evidence suggests that the cGAS-STING signaling pathway, beyond its antiviral functions, also plays a significant role in host defense against bacterial infections. However, its role in antibacterial immunity is complex and context-dependent, varying with the bacterial species, route of infection, and host cell type.

*Staphylococcus aureus (S. aureus)*, a gram-positive opportunistic pathogen, causes a range of infections and poses a major clinical challenge due to antibiotic resistance. While the cGAS-STING pathway is well established in antiviral defense through sensing cytoplasmic DNA, its role in bacterial infections remains complex. In a skin infection model, Philip et al. showed that *S. aureus* released DNA to activate the cGAS-STING pathway, inducing an IFN-I response. However, this response antagonizes the toll-like receptors (TLRs) signaling, reducing the IL-1β production and neutrophil recruitment, thereby impairing host defense and facilitating bacterial evasion (Scumpia et al., 2017). In contrast, in a pneumonia model, STING limited *S. aureus* infection by preventing macrophage necroptosis. STING deficiency led to increased macrophage death, bacterial proliferation, and inflammation, while pharmacologic inhibition of necroptosis restored host defense defects in STING-deficient mice (Liu et al., 2021). These findings highlight a dual role of STING in *S. aureus* infections — either impairing or enhancing host immunity depending on the infection site and immune context.

*Streptococcus pneumoniae* (*S. pneumoniae*) is the leading bacterial cause of community-acquired pneumonia, with high global mortality and morbidity (Drijkoningen and Rohde, 2014). The innate immune system detects the cell-wall components, toxins, and nucleic acids of *S. pneumoniae* through PRR inflammatory responses to combat infection. Among these, cGAS uniquely senses cytoplasmic dsDNA, catalyzing the synthesis of the second messenger 2’3’-cGAMP. In addition to recognizing cGAMP, STING also can directly sense cytoplasmic cyclic dinucleotides (CDNs) like cyclic-di-adenosine monophosphate (c-di-AMP) and cyclic-di-guanosine monophosphate (c-di-GMP), leading to TBK1/IRF-3 activation and IFN-I production (Burdette et al., 2011; Cai et al., 2014). CDNs serve as essential bacterial secondary messengers and are capable of inducing IFN-I responses in monocytes and macrophages. Once internalized via clathrin-dependent endocytosis, extracellular CDNs (eCDNs) bind cGAS directly, leading to its dimerization and promoting the formation of cGAS/STING complexes, further activating downstream signaling (Liu et al., 2019). Therefore, CDNs support both direct innate immune activation and DNA sensing pathways (Liu et al., 2019). Furthermore, STING also regulates coagulation by modulating intracellular calcium levels and promoting the release of key coagulation factors independent of TBK1 or IRF3 activation, thereby mitigating sepsis severity during *S. pneumoniae* infection (Zhang et al., 2020). Pneumolysin (Ply), a major pneumococcal toxin, disrupts host mitochondrial function, triggering mtDNA release and STING activation (Hu et al., 2019). However, *S. pneumoniae* can evade the host’s immune response through specific mechanisms. First, it employs phosphodiesterases Pde1/Pde2 to rapidly hydrolyze self-synthesized c-di-AMP, thereby preventing leakage of CDNs into the host cytoplasm and subsequent activation of the STING pathway (Wooten et al., 2020). Second, EndA endonuclease degrades bacterial DNA and NETs (Zhu et al., 2013), reducing the likelihood of DNA entering host cells and activating cGAS.

*Legionella pneumophila* (*L. pneumophila*) is an opportunistic intracellular pathogen that enters the host via inhalation of contaminated aerosols and replicates within alveolar macrophages, causing severe pneumonia. The host immune system detects *L. pneumophila* through multiple PRRs, including TLRs, NOD-like receptors, and cytosolic DNA sensors such as the cGAS-STING pathway (Opitz et al., 2010; Zamboni and Massis, 2011; Chaput et al., 2013; Brown et al., 2017). These receptors activate immune responses via distinct signaling pathways to restrict pathogen replication. Among these, the cGAS-STING pathway plays a critical role in the host defense by inducing IFN-β, which enhances macrophage bactericidal activity against *L. pneumophila* (Lippmann et al., 2011). In addition, IFN-I upregulates immune-related GTPases (GBPs), facilitating the fusion of Legionella-containing vacuoles with lysosomes, thereby promoting bacterial clearance (Naujoks et al., 2016). Furthermore, IFN-I induces immune response gene 1 (IRG1), which drives the production of itaconic acid, a citric acid cycle metabolite with antimicrobial properties that directly inhibits *L. pneumophila* (Naujoks et al., 2018). However, *L. pneumophila* employs its Dot/Icm secretion system to deliver multiple effector proteins that effectively suppress the activation of innate immune pathways. For example, SdhA plays a critical role in maintaining the integrity of the LCV membrane, thereby preventing host cells from detecting bacterial DNA (Harding et al., 2013). Additionally, SidE-mediated PR ubiquitination specifically targets several ER and Golgi apparatus associated proteins, leading to the disruption of the endoplasmic reticulum membrane, disassembly of the Golgi structure, and subsequent interference with vesicle trafficking and cellular secretion pathways (Xie et al., 2023).

Non-typable Hemophilus influenzae (NTHI) is a prevalent respiratory pathogen. Due to its lack of a polysaccharide capsule, NTHI is not effectively targeted by current vaccines. Upon NTHI infection, the host cell expression of cGAS and STING is markedly upregulated, with STING activation closely linked to IFN-β production. Gene knockout studies further demonstrate that the absence of either cGAS or STING significantly reduces IFN-β expression in response to NTHI DNA (Lu et al., 2018), highlighting the critical role of the cGAS-STING signaling pathway in host response to NTHI. IFN-I plays a dual role in antibacterial immunity. While they enhance the host defense mechanisms, excessive IFN-I signaling can lead to immune-mediated tissue damage. In NTHI infection, IFN-I promotes the expression of chemokines such as CXCL10, facilitating the recruitment of immune cells to the infection site and amplifying local immune response (Proost et al., 2006). However, an overactive IFN-I response may also exacerbate inflammation, potentially worsening underlying conditions like COPD. Current research on the mechanisms by which NTHi directly interferes with the cGAS-STING pathway remains relatively limited. Existing studies have demonstrated that NTHi modifies its lipooligosaccharide (LOS) through phosphorylcholine addition, thereby altering its surface structure and reducing the probability of recognition by the host immune system (Clark et al., 2012). This modification enables NTHi to evade complement-mediated killing and antibody recognition and may also indirectly influence the activation status of the cGAS-STING pathway.

*Chlamydia* is obligate intracellular gram-negative bacteria responsible for various human diseases, including trachoma, reproductive tract infections, and pneumonia. Studies have demonstrated that *Chlamydia* activates the cGAS-STING pathway via two mechanisms (Barker et al., 2013; Zhang et al., 2014): by releasing dsDNA that stimulates cGAS and by synthesizing cyclic dinucleotides (e.g., c-di-AMP) that directly activate STING. This activation triggers multiple downstream immune responses, such as autophagy, IFN-I production, and inflammasome activation (Wu et al., 2013; Webster et al., 2017; Gui et al., 2019). Su et al. found that *Chlamydia trachomatis* infection induces IFN-β production through the cGAS-STING pathway, suppressing bacterial replication (Su et al., 2022). Furthermore, *Chlamydia* infection generates mitochondrial ROS (mtROS), leading to oxidative damage and cytoplasmic release of mtDNA. Subsequently, the cGAS-STING-IRF3/NLRP3-axis is activated, resulting in IFN-I and IL-1β production, enhancing inflammation, and restricting *Chlamydia* replication in macrophages (Yang et al., 2024). However, the role of IFN-I in *Chlamydia* infections remains controversial. While some studies suggest that IFN-I suppress *Chlamydia* growth *in vitro* (de la Maza et al., 1985; Rothfuchs et al., 2001; Ishihara et al., 2005), others suggest it can induce macrophage apoptosis *in vivo*, weakening the host’s innate immune response and exacerbating infection (Qiu et al., 2008).

*Pseudomonas aeruginosa* (PA), a gram-negative opportunistic pathogen, can cause severe nosocomial infections, primarily affecting the lower respiratory tract, surgical and wounds, urinary tract, and cornea (Priebe and Goldberg, 2014; Yang et al., 2016; Huang et al., 2019). Its pathogenicity is notably elevated in immunocompromised individuals (Wilhelmus, 1987). Although primarily an extracellular bacterium, PA DNA can enter host cells and activate the cGAS-STING pathway. Through gene knockout experiments, Zhou et al. confirmed that cGAS and STING play protective roles against PA-induced pulmonary infections (Zhou et al., 2021). The cGAS-STING-IFN-I axis is essential for innate immunity, as it regulates IFN-I signaling while modulating ER stress and activating the unfolded protein response (UPR) (Zhou et al., 2021). The UPR is vital for host defense against pathogens (Richardson et al., 2010; van ‘t Wout et al., 2015) and is closely associated with the cGAS signaling pathway (Moretti et al., 2017; Guimaraes et al., 2019; Wu et al., 2019). During PA-induced UPR, IRF3 was shown to directly bind to the promoter regions of BIP and ATF4, promoting the transcription of UPR-related genes (Zhou et al., 2021). Host clearance of PA relies on multiple bactericidal mechanisms, including nitric oxide (NO)-mediated oxygen-dependent pathways. STING enhances antibacterial activity by upregulating inducible nitric oxide synthase (iNOS) expression and boosting NO production (Chen et al., 2018). In addition, STING modulates key inflammatory signaling pathways. It inhibits NF-κB nuclear translocation, thereby reducing IL-1β, IL-6, and TNF-α levels and limiting immune-mediated tissue damage (Chen et al., 2018). STING also regulates the MAPK signaling cascade—particularly the p38, JNK, and ERK pathways—further highlighting its role in inflammation during infection (Chen et al., 2018; Haj et al., 2025). Despite the lack of direct research on the immune escape mechanisms of Pseudomonas aeruginosa targeting the cGAS-STING pathway, existing studies indicate that exotoxins secreted by Pseudomonas aeruginosa, such as ExoU, can induce cell death through membrane disruption, thereby indirectly attenuating the activation of the cGAS-STING pathway (Hardy et al., 2021).

*Mycobacterium tuberculosis* (*M. tuberculosis*), the causative agent for tuberculosis (TB), remains one of the deadliest infectious pathogens worldwide (Chai et al., 2018). The key virulence determinant factor of *M. tuberculosis* is the ESX-1 secretory system, which disrupts host membranes, releasing mitochondrial and nuclear DNA into the cytoplasm. This, in turn, activates the cGAS-STING pathway and promotes IFN-I production (Lienard et al., 2020). Further, *M. tuberculosis* synthesizes c-di-AMP, a PAMP that activates STING and induces IFN-β via IRF3, independently of cGAS, thereby enhancing macrophage-mediated bacterial clearance (Dey et al., 2015). However, *M. tuberculosis* employs multiple immune evasion strategies to interfere with the cGAS-STING signaling pathway. One such mechanism involves the bacterial protein Rv0753c (MmsA), which promotes STING degradation via p62-mediated autophagy, thereby dampening immune activation (Sun et al., 2020). MmsA also suppresses IFN-I signaling upstream of IRF3 activation (Sun et al., 2020). Furthermore, clinical isolates from patients with severe TB exhibit mutations affecting the ESX-1 system, allowing evasion of cytoplasmic immune sensors, such as cGAS and inflammasomes, leading to reduced IL-1β production and diminished host immune response (Sousa et al., 2020). Another evasion mechanism involves the bacterial enzyme cyclic di-nucleotide phosphodiesterase (CdnP), which hydrolyzes both bacterial c-di-AMP and host-derived 2’3’-cGAMP, thereby inhibiting STING-dependent IFN-I signaling (Dey et al., 2017). These diverse strategies enable *M. tuberculosis* to persist within the host and contribute to the chronic nature of TB. Understanding how *M. tuberculosis* manipulates the cGAS-STING pathway provides valuable insights for developing novel TB vaccines and therapeutics. Modulation or enhancement of the cGAS-STING pathway may strengthen host immune defense, alleviate TB symptoms, and improve treatment outcomes.

## 4 The role of cGAS-STING pathway in modulating antifungal immune responses

*Aspergillus fumigatus* represents a clinically critical fungal pathogen, predominantly responsible for invasive pulmonary aspergillosis (IPA) — a severe fungal infection characterized by elevated rates of morbidity and mortality (Margalit and Kavanagh, 2015; Hage et al., 2019; Koehler et al., 2021). While cGAS-STING signaling axis has been extensively characterized for its role in antiviral and antibacterial host defense mechanisms, its contribution to antifungal immune responses remains poorly understood. Notably, recent investigations conducted by Peng at al. have elucidated that cGAS-mediated signaling enhances innate immune responses against *A. fumigatus*, thereby conferring protective immunity to the host. Specifically, activation of the cGAS-STING cascade facilitates efficient clearance of fungal pathogens, whereas its impairment not only exacerbates inflammatory responses but also undermines antifungal immune defenses (Peng et al., 2023). However, the clinical management of Aspergillus fumigatus infections remains a significant challenge. The RodA hydrophobin on the surface of Aspergillus fumigatus spores forms a protective “rodlet layer”, which masks cell wall-associated PAMPs such as β-glucan, thereby evading recognition by host PRRs and dampening immune responses (Carrion et al., 2013). Gliotoxin (GT), a secondary metabolite secreted by Aspergillus fumigatus, exhibits potent immunosuppressive properties. It induces apoptosis in macrophages and neutrophils and inhibits NF-κB signaling, leading to reduced production of inflammatory cytokines (Günther et al., 2024).

## 5 Prospects

The cGAS-STING pathway, a critical component of the innate immune system, plays a central role in antiviral defense, cancer immunotherapy, autoimmune conditions, and neurodegenerative disorders. With a deeper understanding of its molecular mechanisms, the potential applications of the cGAS-STING pathway in therapeutics are becoming increasingly evident. For instance, a novel X-ray-inactivated PA whole-cell vaccine induces the release of bacterial nucleic acids, activating the cGAS-STING signaling in dendritic cells and subsequently inducing pathogen-specific T-cell responses, thereby enhancing host resistance to infection (Ma et al., 2021). Furthermore, insights into the immune evasion mechanisms employed by pathogens against the cGAS-STING pathway provide critical insights into developing immunomodulatory therapeutic strategies. Significant progress has been made in designing therapeutics targeting this pathway (Haag et al., 2018; Pan et al., 2020; Hong et al., 2021). STING agonists, including cGAMP analogs, show promise in cancer immunotherapy and as vaccine adjuvants, while small-molecule inhibitors of cGAS or STING, such as covalent STING inhibitors, demonstrate potential for treating autoimmune diseases (e.g., AGS) and other chronic inflammatory disorders. Despite these advances, translating such therapies into clinical practice remains challenging. Excessive activation of cGAS-STING can lead to chronic inflammation or autoimmune responses, necessitating precise control of its activation level. In addition, improving drug delivery systems to enhance targeting and stability, addressing individual variability, and ensuring safety are key considerations for future studies.
